# Doing primary care integration: a qualitative study of meso-level collaborative practices

**DOI:** 10.1186/s12875-023-02104-7

**Published:** 2023-07-17

**Authors:** Myles Leslie, Raad Fadaak, Nicole Pinto

**Affiliations:** 1 School of Public Policy, University of Calgary, 906 8 Ave SW 5th floor T2P 1H9, Calgary, AB Canada; 2 Cumming School of Medicine, Department of Community Health Sciences. 3D10, University of Calgary, 3280 Hospital Drive NW Calgary, Calgary, Alberta, Alberta T2N 4Z6 Canada

**Keywords:** Primary care, Primary health care, Integration, Meso-level, Collaboration, Qualitative study

## Abstract

**Background:**

The integration of Primary Care (PC) into broader health systems has been a goal in jurisdictions around the world. Efforts to achieve integration at the meso-level have drawn particular attention, but there are few actionable recommendations for how to enact a ‘pro-integration culture’ amongst government and PC governance bodies. This paper describes pragmatic integration activity undertaken by meso-level participants in Alberta, Canada, and suggests ways this activity may be generalizable to other health systems.

**Methods:**

11 semi-structured interviews with nine key informants from meso-level organizations were selected from a larger qualitative study examining healthcare policy development and implementation during the COVID-19 pandemic. Selected interviews focused on participants’ experiences and efforts to ‘do’ integration as they responded to Alberta’s first wave of the Omicron variant in September 2021. An interpretive descriptive approach was used to identify repeating cycles in the integration context, and pragmatic integration activities.

**Results:**

As Omicron arrived in Alberta, integration and relations between meso-level PC and central health system participants were tense, but efforts to improve the situation were successfully made. In this context of cycling relationships, staffing changes made in reaction to exogenous shocks and political pressures were clear influences on integration. However, participants also engaged in specific behaviours that advanced a pro-integration culture. They did so by: signaling value through staffing and resource choices; speaking and enacting personal and group commitments to collaboration; persevering; and practicing bi-directional communication through formal and informal channels.

**Conclusions:**

Achieving PC integration involves not just the reactive work of responding to exogenous factors, but also the proactive work of enacting cultural, relationship, and communication behaviors. These behaviors may support integration regardless of the shocks, staff turnover, and relational freeze-thaw cycles experienced by any health system.

**Supplementary Information:**

The online version contains supplementary material available at 10.1186/s12875-023-02104-7.

## Introduction

Integration of healthcare services has been a policy goal in jurisdictions around the world for decades [1, 2]. Specifically, the integration of primary care (PC) into broader health systems and social services has been a focus [3–6], with PC positioned as a key access and coordination point in a broader move to deliver Primary Health Care (PHC) [7–11]. Efforts to achieve integration have approached it as an end state in which diverse clinicians come together to provide care to those with complex health needs, while eliminating redundancies or gaps in service [12]. From this broad and clinically focused definition, a literature has developed that studies integration as it is attempted at micro-, meso-, and macro-levels [13].

The present paper analyzes the meso-level integration activities of PC participants as they sought to accomplish more by collaborating across the organizational silos of their health system [14, 15]. Meso-level here is distinct from both the clinical operations of the micro-level, and the macro-level efforts of national or international organizations seeking high-level policy changes. We describe the integration efforts of meso-level participants from implementation-oriented departments of the government, operations-focused PC governance organizations, and associations or colleges representing PC providers in Alberta, Canada. We describe them attempting to ‘do’ integration at a time when relationships were tenuous, and at a particular moment in the COVID-19 (C19) pandemic. By showing a particular group of PC participants signaling one another, sharing ways of thinking, and enacting cultural values [16, 17] and so achieving greater continuity of planning and care delivery in the pandemic response [18], our aim is to make a broader contribution to the practice of integration.

While a robust body of literature describes the facilitators of (and barriers to) integration at the micro-level [19–21], the effects of local culture on the uptake of integration-focused improvement initiatives remain ‘underspecified,’ [22].Similarly at the meso-level, the evidence available to support actually doing complex culture change [23, 24] has been described as “thin” [25]. This is to say the literature often invokes high-level concepts like “culture”, “relationships”, or “communication” as important factors, but rarely examines the ways in which these concepts are enacted, particularly as facilitators between institutions or organizations.

In the analysis that follows, we illustrate how participants practically enacted culture, relationships, and communications in a way that fostered integration. Our work here aligns with that of PC scholars who have sought to understand integration as an emerging set of practices [18]. Specific mechanisms of integration, such as quality improvement measures [26] performance feedback [27], incentives and financing models [13, 27–30] and governance structures to promote accountability [5, 13, 26, 31] have been identified. Across these mechanisms, and the micro-, meso-, and macro-levels of intervention, the literature suggests the importance of establishing a pro-integration culture [32] that emphasizes inter-organizational relationships [13, 26], and facilitates open communication [27, 33]. Beyond these high-level encouragements to pursue underspecified goals, the evidence for how to go about this culture, relationship, and communication work is thin. What follows draws pragmatic lessons in ‘doing’ meso-level integration from the experiences of Alberta’s PC participants.

## Background

PC in Alberta is directly financed by the province’s ‘single payer’ [34, 35] Ministry of Health (MoH), and most care is delivered by independent family physicians who bill the government on a fee for service (FFS) basis. The fees a family physician may charge are negotiated between the Alberta Medical Association (AMA) and the MoH. Over the last decades, remuneration, the viability of PC practices as businesses paying their own overhead, and efforts to ensure access to PC for increasingly complex patients in a range of settings across the province, have made those negotiations contentious, while also leading to significant innovation [33]. We describe this innovation in more detail below.

Alongside the independent FFS model of PC delivery, the province operates the largest centralized healthcare system in Canada, with over 650 facilities managed by a single health authority: Alberta Health Services (AHS). AHS is charged by the MoH with delivering care in five geographically-based ‘health zones,’ with facilities providing acute, long-term, and urgent care. A small PHC-focused unit inside AHS is devoted to achieving PC integration as a policy objective [36]. In addition, innovative bridging organizations called Primary Care Networks (PCNs), financed through capitation, act as key points of contact between central AHS and independent family physicians who opt-in to PCN membership [33].

As C19 arrived in Alberta in March of 2020, relations between the MoH and the AMA were cold. The negotiation of virtual care billing codes to facilitate community management of C19 patients took place at a moment of deep chill in the relationship between independent PC and the central system [37–39]. Nonetheless, over time, the relationship warmed and mutually acceptable arrangements were made [5]. The data we present below describe activity at a subsequent moment in what we argue is an ongoing, and perhaps universal, cycle of freezing and thawing relationships in the integration context.

## Methods

An ‘interpretive description’ approach [40] was taken to analyse interviews with key informant participants. Interpretive description focuses on identifying applied knowledge and allows for specific attention to participants’ institutional commitments and perspectives [41, 42]. It provides insights not just into areas of commonality but also areas of disagreement among participants, with an eye on unearthing pragmatic suggestions to improve policies and outcomes [40, 43]. With research showing that purposive, rather than random, sampling is effective at drawing information from small samples [44, 45] focused interviews with a limited number of key informants were conducted. This approach has been validated as increasing the likelihood of attaining data saturation [46], with evidence indicating that when a qualitative study: is more specific in its aims; targets informants with specific knowledge; and relies on interviews of sufficient length and quality, saturation can be achieved with relatively low sampling [47].

Semi-structured qualitative interviews with key informants were conducted as part of a broader project seeking to understand the experiences of healthcare participants from across the provincial health system as they responded to C19. Participants in the broader project were initially recruited purposively based on their administrative or clinical roles and involvement in the creation or implementation of pandemic response policies. A snowball sampling strategy was adopted thereafter as we identified areas of policy innovation and implementation activity. Our particular focus was on the challenges encountered as policy was implemented across the boundaries of health system organizations [removed for peer review purposes] [48]. In this way administrative, physician, and patient leaders from a range of micro-, meso-, and macro-level organizations were interviewed in the full sample, with data collection focused on moments of policy transfer or implementation that spanned boundaries or sought to coordinate and integrate activity [removed for peer review purposes] [49, 50].

From that full sample of semi-structured interviews (n = 127) with 114 unique participants, the present analysis focuses on 11 interviews with nine key informants from meso-level organizations conducted by [Removed for Peer Review Purposes] between January and March 2022. The focus of these interviews was on the planning for, and response to, the Omicron wave which began in September of 2021. The Omicron wave was, in this sense, an episode identified in the broader sample of interviews that could be analyzed to identify themes and draw lessons in doing integration at the boundary between PC and the central system. While anonymity concerns preclude us from offering details about the meso-level organizations that these key informants were associated with, Table 1 provides an overview of their alignment with either independent PC or central-system aspects of the integration context.

**Table 1:** Key Informant Interview Participants in *Methods*

Participant No.	Primary Affiliation	General Descriptor
018	Central System	Physician Leader
074	Central System	Administrative Leader
079	Central System	Physician Leader
109	Independent PC	Administrative Leader
110	Independent PC	Physician Leader
111	Central System	Administrative Leader
112	Independent PC	Physician Leader
113	Independent PC	Physician Leader
114	Central System	Physician Leader

The interviews in the present paper averaged 65 min in length and were digitally recorded and transcribed for analysis. [Removed for peer review purposes] analyzed the data to identify themes. Analysis was supported by MAXQDA 2022 software and deployed an inductive coding approach to render an interpretive description of key informants’ integration experiences and activities at the boundary between PC and the central health system. The authorial team analyzed the data iteratively, expanding, collapsing and merging themes to arrive at the final analysis. We summarize passages from the verbatim transcripts in our analysis and provide access to the full quotes in Boxes at the close of each section in the Results. We attribute the quotes to participant numbers ranging from 18 to 114 (see Table 1). This research obtained ethical approval from the Conjoint Health Research Ethics Board at the University of Calgary (REB20-0371). All participants provided written and verbal consent.

## Results

### Contextual freeze-thaw cycle

Participants felt the pandemic had catalyzed significant connectivity across the PC environment, increasing interactions between the various parts of the health system (Table 2, Quote 1). However, relations between the MoH and PC were decidedly cool in the summer of 2021. MoH officials were taking direction from a government that all-but declared the pandemic over as it vowed to make the season the ‘best ever’ [50, 51], and PC leaders felt they had been taken advantage of. Just a week before, they had begun work on a plan for delivering PC under endemic conditions, and were caught off guard by government moves to suspend mass testing and return to ‘normal’ (T2, Q2). Beyond surprised, PC leaders felt snubbed, betrayed, and like the engagement and consultation process to integrate PC into the ongoing pandemic response was disingenuous (T2, Q3). Some acted on these feelings by walking away from the conversation entirely (T2, Q4). In a contextual cycle of freezing and thawing relationships between the MoH and PC, then, June 2021 marked a particularly cold point and a low in meso-level PC integration into the pandemic response.

**Table 2:** Verbatim quotes supporting *Freeze-Thaw Cycle*

Quote No.	Partici-pant No.	Quote
1	074	[The pandemic saw] the development of new relationships. And not just with AHS-PHC and the MoH, but with a whole bunch of other groups that you certainly would not have worked with before, which is of huge benefit.
2	113	[Independent PC, and AHS-PHC leaders] were engaged by [the MoH to start planning] – we were thinking September, October – for an endemic environment. We were one week into our [June] conversation, [and the MoH] declared that at the end of that month, they would: switch all [COVID-19] testing to primary care; discontinue testing in AHS facilities, and basically move on into a new environment. That caught us completely off guard.
3	112	[The government was] already committed [to COVID-19 being] endemic. And so [the consultation with PC was] a rear guard action [where they were saying,] “Here’s some [rapid antigen] test kits. We’ll throw them at you. And hopefully that helps a little bit.”
4	079	[Some PC leaders] walked away from the table, [saying] “*Expletive* yourself! If you’re not going to listen to me and you’re not even going to take what I’m saying seriously, and then you’re going to lie about having consulted with me in public, I’m not even showing up anymore.”
5	074	[There’s] a looming election. [And the government starts to think] “we have to strategically [put] another minister in there because of the destroyed relationship [with PC] and our need to actually move forward together.”
6	109	[In the autumn] we’re under the watch of a new minister who has set a very different tone. Who has engaged directly not just with [the AMA] President, but has asked for the President to arrange briefings by physicians from various sections, including primary care and rural care.
7	113	When the minister changed … it was in rapid succession thereafter that things started getting better. [That’s also] when [the new Assistant Deputy Minister] came into the picture.
8	110	Suddenly [the new Deputy Medical Officer of Health] was here, and we sort of heard about it, and she has seemed to reconvene the collaborative table where we’re all working together on [the newly arrived Omicron variant], and she [proves to be] a source of good information and consultation.

Months later, however, the cycle reversed. According to participants, the thaw in relations, and so increase in PC integration, was attributable to a range of exogenous factors: (1) The arrival of the Omicron variant that defied the optimism of the summer; (2) A provincial cabinet shuffle (T2, Q5) introduced a new consultative style (T2, Q6); (3) A new Assistant Deputy Minister at the MoH took up the health portfolio and made good on the new Minister’s consultative style (T2, Q7); and (4) The hiring of a new Deputy Chief Medical Officer of Health (DCMOH) who brought the various participants back to the table, provided reliable information, and lived the consultative ideal (T2, Q8). Under these conditions, as the ‘best summer ever’ became the autumn of Omicron, a freeze-thaw cycle in Alberta’s integration context moved into a warmer period.

### Doing pro-integration culture

Achieving a pro-integration culture involved sending value signals through staffing choices; personal expressions of commitment to the values of integration; and ensuring communal commitments to those values. The hiring of a new DCMOH – at the request of an overstretched Chief Medical Officer of Health (Table 3, Quote 1) – was a key moment in the thaw. The Chief sent a signal by creating a position focused on PC integration and hired someone who was able to deliver on her priorities and consultative style (T3, Q2).

**Table 3:** Verbatim quotes supporting *Doing Pro Integration Culture*

Quote No.	Partici-pant No.	Quote
1	114	[The Chief Medical Officer of Health] certainly felt that she was unable to be that consistent presence, and create that connection with primary care and with physician colleagues. Yet it was a priority for her. But given her schedule and demands on her time, she kept being called away and unable to meet. So she asked [the new DCMOH] to do that on her behalf, [and to connect with the MoH PC team].
2	109	There’s a new physician lead [hired and] one of [their] jobs is to confer with primary care. And they start doing that, they start conferring!
3	74	If you want to truly engage and get the most out of those engagements, [you have to] have the right players to the table. So, a champion within that particular group you’re trying to move … So in this case, you’re trying to engage family physicians, you actually have to have a champion family physician.
4	110	It just feels better because we’ve got an advocate in the office of the Chief Medical Officer of Health. And I think [the MoH primary care unit] is more willing to be collaborative… [On the ground having that advocate means that] I feel very comfortable firing off an idea for expedited return to work, for instance, in primary care offices to [the new DCMOH, and asking them], “Does this make sense with your guys’ public health order?” I would never have done that with the Chief. I would just go, “Oh, she’s too busy.”
5	114	It’s been a huge investment as well from AHS, they’ve devoted lots of their staff time to help make this happen. Because physicians are busy people, and they don’t have a lot of hours left in the day, and so it really has been, I’d say, a very strong team effort across various parts, whether it’s primary care or AHS or [the MoH]. We’re trying to work to each other’s strengths, and contribute in whatever way we can to make this happen.
6	112	[Since the new DCMOH arrived] you see the messaging, you see the open attitude of collaboration to start with, and you see that your input is making a difference in actions and decision-making. That builds trust over time. [You see the DCMOH saying:] “Hey guys, this is an issue we’re looking at. This is what we’re thinking. Are we missing anything? Are there any red flags here? What else do we need to know? What other topics are we needing to discuss?“ [They just] come in with a collaborative attitude.
7	18	I think there have been a couple of changes within the MoH where some people are a little more interested in listening and not assuming that there’s an agenda behind the advice [that we in PC might be giving.] Which was the feeling that we often had before. [It was like they thought:] “If you’re a physician giving advice, you have some hidden agenda related to compensation or something like that.” So that feels like that’s thawed a bit.
8	079	[the new DCMOH] just wants to work with us and doesn’t shut us down all the time.
9	110	we are working towards a common goal.
10	113	[When the MoH said], “Okay, now let’s bring people to the table, and let’s actually start playing less directive and more collaborative,“ that’s when things started changing. I think [that built] trust at a bureaucratic table, [as] the minister [of health said] “You know what, let’s be more collaborative.”
11	113	[It was like they realized] if this is going to work, we need [the MoH], AHS and primary care…to co-chair [the work] so that we all have a stake in the game. And we also need to be more open to involving other groups in the process.
12	110	[Expanding the table became a matter of finding] maybe not the traditional leaders to integrate into the system, but maybe those people that are working on the workarounds of the system. I think a lot of people spend a lot of time just working around the policies that exist to make solutions that make sense for their patients, and [there was a realization that] it would be really great to have a table to voice that.

This signal that PC was valued was received as intended in both the PHC-focused unit inside AHS and on the front lines of PC. The AHS-PHC unit saw the choice to hire a family doctor into the DCMOH role as a step towards legitimate, good-faith engagement (T3, Q3), and leaders in the independent PC world felt, with the appointment, that they had an easily-engaged advocate in the halls of power (T3, Q4). Similar staffing choices, and so signals of the value of integration work, were sent by the AHS-PHC unit. These were also well received, creating a sense of team and collective enterprise (T3, Q5). Thus, doing integration involved successfully sending a value signal through staffing and resource choices. The values of pro-integration culture were made real through the creation of specific jobs and a commitment to integration work.

Personal expressions of commitment to integration were similarly seen as enactments of a shared value and common priority. Expanding on the importance of consultation, PC leaders emphasized how words and deeds that showed personal commitments to integration built trust over time (T3, Q6). Specific actions beyond being consultative and collaborative (T3, Q6) included: assuming good faith and intentions (rather than bad-faith and selfish intent) on the part of others (T3, Q7), and indicating a desire to work together by not shutting others down (T3, Q8).

In addition to individual signals of commitment to the collaborative values of pro-integration culture, participants also described communal expressions through shared work. A PC leader used a collective pronoun to describe how the work of the autumn thaw period felt like the various participants were striving for common goals (T3, Q9). Another PC leader invoked collaboration, contrasting it with directive command and control approaches, as the enactment of integration’s cultural values. They also noted that in ‘playing more collaborative’ and ‘being less directive,’ their MoH counterparts were engendering trust (T3, Q10). Trust here was accomplished by the assumption of good faith noted above, and by the inclusion of more voices and perspectives (T3, Q11-2).

### Doing integration relationships

Working at the relationships that contributed to, or were able to benefit from, a thawing integration environment required participants to follow through on their rhetoric, and persevere in proving themselves to their counterparts. From the perspective of both MoH and PC leaders, following through on stated intentions was key to doing the relationship work that supported integration. As part of enacting the pro-integration value of collaboration, all those involved needed to do what they had promised (Table 4, Quote 1). PC leaders judged their MoH counterparts’ follow through work against real-world outcomes. Those outcomes might include changed public health messaging that incorporated PC perspectives; or a willingness to revisit the billing codes that kept independent PC financially viable (T4, Q2); or supplying government purchased personal protective equipment to PC practices (T4, Q3). This was not to say PC leaders demanded follow-through on all their issues, but rather that doing the relationship work of integration required an openness to discussion and making good on commitments (T4, Q4). Following through, then, was a key to enacting a healthy integration relationship.

**Table 4:** Verbatim quotes supporting *Doing Integration Relationships*

Quote No.	Partici-pant No.	Quote
1	114	I think it’s about following through on what you say you’re going to do. Holding each other accountable. Chipping in and helping and [finding] the solutions. Work[ing] together towards common goals. And it’s following through on that. It’s been a very successful collaboration; that’s because people are committed, and they’re doing what they said they’re going to do.
2	112	[You see follow through when] you see that your input is included in messaging and decision making. And things happen…that actually work to meet your needs. The minister being willing to look at virtual [billing] codes and making changes there in the absence of an agreement is also [an example].
3	109	[In the Autumn thaw period] the engagement was different, so the result was different. We were able to raise things like, “[Sourcing] Personal Protective Equipment [in PC] is going to be a problem.” [And the MoH] said, “Right, we’ll take that away, we’ll see what we can do.”
4	110	It doesn’t mean [the MoH always] acts on [your input], but there seems to be a more open discussion.
5	113	I would say you continue to grind [away at the work] in any case, but during the thaw period you probably can make more progress. During the freeze period, you’re not going to stop working and stop trying. [You need to] show resilience and [try] to forge forward.
6	074	[During the freeze] I think [the relationship] publicly looks dormant. I don’t think it is [you have to keep working at it]. And then you can set it aside, that ugly relationship, [when the thaw comes.]
7	113	We’ve fought and we struggled so long, so hard, to be recognized, to be valued. There was probably not a better opportunity for [PC] to show our value than with [COVID] and [prove] our impact on the healthcare system. That’s the motivator that continued to drive us [in the freeze period].
8	079	I think if there’s one hero character to all of this: it’s [participant 113 and how they] kept selling [PC to the MoH]
9	113	When Omicron came [in the autumn and relations thawed], we took the same terms of reference with exactly the same recommendations that we made [in the spring during the freeze]. And I showed [them] to the new ADM and I said, “You want the solution? We had the solution four months ago. Here’s your solution.“ And with him reading through that, I think something sparked because the whole attitude of [the MoH] changed. So I actually think that [was the moment the MoH started to] trust [us].

Similarly, participants noted that perseverance in the integration relationship was important. As a PC Leader described it, perseverance was a steady ‘grind’ of relationship work that, even if it yielded more success in a thaw period, required effort even during cooler periods (T4, Q5). An AHS-PHC leader described how perseverance bridged the freeze periods, setting the possibility for integration when the thaw came, and previous misgivings could be set aside (T4, Q6). This characteristic of perseverance was described as an ongoing struggle to have PC recognized and valued (T4, Q7) and a heroic undertaking in the face of frozen relationships (T4, Q8). Through perseverance, despite the odds and the summer’s freeze, PC leaders were able to immediately take advantage of the autumn’s thaw. They could take not just ideas, but fully formed plans that had been rejected earlier, and present them to newly attentive eyes (T4, Q9). Being present and seeking constantly to prove the value of PC in the pandemic response were essential to enacting integration. Persevering with relationships that might look dormant, or worse, was of central importance.

### Doing integration communication

Participants described specific ways of communicating to achieve integration. These included: practicing bi-directional communication; and actuating a mix of formal and informal communication channels. Practicing bi-directional communication was a key behavior underpinning the thaw in relations that all participants described. As parties on both sides showed they were willing to listen, as well as to talk, the communication pattern was perceived both as more cordial (Table 5, Quote 1), and as having shifted from uni-directional commands from the MoH, to a reciprocal flow of co-designed ideas and feedback from PC on implementation efforts (T5, Q2). The shift was seen as central to achieving two-way consultation, and so the integration of PC into policy formation (T5, Q3).

**Table 5:** Verbatim quotes supporting *Doing Integration Communication*

Quote No.	Partici-pant No.	Quote
1	74	[In the autumn there was] a change of [leadership] and now this is somebody who doesn’t come with the same baggage, who is willing to listen and talk. So it becomes cordial.
2	110	[PC leaders can now give] opinions so that [the MoH is] not just flying blind and telling us what they’re going to do, [but] we can give back some information.
3	111	[I call it] the sausage-making [table]…We created a forum for two-way consultation. [The MoH] can take things to [PC leaders], just to get their feedback. And it also helps them give us [ie. the MoH] information, and ask for things that we can take forward as well. So it’s helpful. [And we’re asking ourselves] “How do we engage other partners, whether it be community physicians or AHS, or eventually it’ll be other ministries? How do we engage with people in terms of being able to bring their advice forward into creating policy?
4	109	[In the autumn] suddenly, the MoH is setting up not one, but *two* committees to work with us. There’s other types of consultations going on informally [as well]. The gates have opened up!
5	110	[With the arrival of the] new health minister, negotiations were going better. The unofficial negotiations [particularly].
6	110	I have that informal relationship [with other PC leaders in other organizations where] we are working towards a common goal. I can talk to [physicians in the AHS-PHC unit] and say “[My organization] is going to do [a] webinar, do you guys want to [come and] speak about what you’re doing?” And so we’re just working closer. And it’s informal. It’s not like we have to do those things.
7	074	[Since the autumn, I think the MoH leaders] truly understand, and maybe were naive to, or underestimated the need for, strong relationships and what [those informal relationships] can actually produce.

A final practical step for achieving communication that supported integration involved accessing a mix of formal and informal channels. While the autumn thaw was characterized by the creation, or resurrection, of formal committees, (T5, Q4) bi-directional consultative communications also occurred in unofficial conversations (T5, Q5). These informal channels took advantage of individual PC leader’s broader social and professional networks, drawing more perspectives and ideas into the relationships that were being cultivated (T5, Q6). Participants emphasized the importance of informal social relationships that extended beyond the formal committee work, describing how those relationships and communication channels were central to achieving integrative goals (T5, Q7).

## Discussion

Our data suggest there is an ebb and flow of people and relationships in the meso-level integration context. Following our participants, we have deployed a temperature metaphor to describe this pattern, calling it a ‘Freeze-Thaw’ cycle. At one level this cycle is the product of factors exogenous to the integration effort, like the C19 Omicron wave, or imposed changes in leadership. At another level, and borne out by our data, organizations can do and learn more about integration beyond relying on exogenous shocks like pandemics to shake things up and alter the ‘temperature.’ In Alberta, the pandemic highlighted pragmatic temperature-raising activities that supported meso-level integration.

The relationships between meso-level organizations in the integrated care literature tend to be conceptualized as more or less deep. Greater depth in those relationships is in turn seen as allowing the organizations to pool skills and expertise and so achieve integration and improve care [13, 51, 52]. Our data, described through a temperature metaphor, suggest this depth varies cyclically. Whether integration relationships are freezing and thawing, or ebbing and flowing, further research is required to understand if and how the cycle presents itself across jurisdictions. While we can be confident that publicly funded health systems are influenced by exogenous factors such as elections, leadership turnover, or pandemics [5, 53], the way these factors shape meso-level participants in varying locations as they ‘do’ integration is unclear. For now, and assuming some level of generalizability from our key informants’ experience of Alberta’s Omicron Freeze-Thaw, we turn to providing pragmatic options for those in other jurisdictions to consider.

The literature on integrating PC into health systems emphasizes the importance of participants espousing a pro-integration culture [32] that focuses on strong relationships [13, 26] and open communications [27, 33]. However, practical advice on doing this culture – enacting its relationships and communicating its values – is thin [54]. Culture, generally, can be defined as the negotiation and expression of commonly held values and norms [55]. In the specific case of a ‘pro-integration culture’ those values and norms have been specified [56], and their negotiation and expression involve community members: visibly manifesting them; sharing ways of thinking; and sharing deeper unsubstantiated assumptions about how integration works [16, 17].

Our data suggest that making pro-integration culture real requires sending and receiving signals that a collaborative working environment is valued. These signals can be sent through: staffing and other resource decisions; matching rhetorical commitments with collaborative actions; shifting from uni-directional talking to bi-directional talking and listening; and activating a mix of formal and informal communication channels. These visible manifestations of individual and group commitments to collaboration are the product of more than a mere attitude switch. From walking the talk to engaging in ‘active listening’ [57, 58], these are enactments of shared mental models [59–61] and the trust that becomes possible when one assumes others are acting in good faith, and when one relinquishes a measure of control over the conversation by adding voices to it. Beneath the visible signs of commonly held values, then, Alberta’s meso-level participants formed what the literature refers to as ‘moral community,’ holding one another mutually accountable for following through on the rhetoric and ideals of collaborative integration culture [62].

The success of this moral community and the flourishing of this pro-integration culture were founded in trust. Trust is characterized by vulnerability [63]. To trust someone is to be vulnerable to them without proof or assurances that they will do what we hope they will do [64]. In this sense, Alberta’s MoH participants made themselves vulnerable to their PC counterparts when they began assuming good faith and positive intent [65], rather than bad faith and selfish intent as had prevailed during the preceding freeze of the contextual cycle. The doctors *might have been* acting in bad faith, but the administrators chose to trust their intentions. Similarly, the MoH participants exhibited vulnerability when they increased the size and scope of the consultation table, thereby relinquishing some of their control of the conversation. Embracing these sorts of vulnerability generated trust. As such, embracing vulnerability appears to be the sort of practical action that can be taken to advance integration. Perhaps even more importantly, those seeking to do integration may wish to consider the value of perseverance. Our data suggest that persevering – at sending the right signals, at maintaining relationships, at active listening as well as talking, at fostering formal and informal communications, at embracing a vulnerability that cedes control but gains trust – is central to achieving integration. It is the quality, applied to all these pragmatic behaviours, that appears to support warming and prevent cooling in the integration context.

## Conclusions

Achieving PC integration likely involves more than passive resignation to the effects of exogenous and contextual factors. The proactive work of enacting specific cultural, relationship, and communication behaviors also appears to contribute. Those seeking to integrate at any given moment in their own context may consider a range of practical activities that enact culture and engender trust: from sending signals with staffing, to connecting rhetoric with demonstrable action; from showing vulnerability to achieve trust, to persevering at relationships. Enacting these visible behaviors, and so the collaborative values that subtend them, appears to support integration regardless of the external shocks, internal staff turnover, and relative coolness of the local freeze-thaw cycle.

## Electronic supplementary material

Below is the link to the electronic supplementary material.

Supplementary Material 1

## Data Availability

The datasets generated and/or analysed during the current study are not publicly available to protect the anonymity of our study participants, but may be available from the corresponding author on reasonable request.

## List of Abbreviations

AHS: Alberta Health Services – the province’s single healthcare delivery authority
AMA: Alberta Medical Association – the province’s medical professional association
DCMOH: Deputy Chief Medical Officer of Health
FFS: Fee for Service – the predominant payment model for primary care in Alberta
MoH: Alberta Health (the province’s ministry of health)
PC: Primary Care
PCN: Primary Care Network
PHC: Primary Health Care (as defined by the WHO)
