# Supplementary material for: Doing primary care integration: a qualitative study of meso-level collaborative practices
Source: BMC Prim Care. 2023 Jul 17;24:149. doi: 10.1186/s12875-023-02104-7 (PMC10353261; doi:10.1186/s12875-023-02104-7)
Supplement: Supplementary file 1 — Supplementary Material 1 [file 12875_2023_2104_MOESM1_ESM.pdf]

Semi Structured Interview Guide

Work through informed consent instrument with participant

Provide participant with signed copy of consent instrument

1. Please describe your organizational affiliation and your current role(s) in your primary organization and any other.

2. Have any of these roles been created or modified with the arrival of COVID-19?

3. Can you describe any other changes in
   - Policy
   - Operations
   - Other

   that have occurred as a result of COVID-19? For each of the above, who initiated the change and/or where did it come from?

4. Please describe **inbound** communications regarding
   - sitreps,
   - case identification,
   - surveillance data collection,
   - testing,
   - supplies,
   - staffing,
   - IPC,
   - Other

   Who has been your counterpart(s)?
   Are there gaps, timeliness, credibility/truth issues with any of these communications?
   How do they mesh (or fail to mesh) with existing or previous operations and plans?

5. Please describe **outbound** communications to
   - Individual patients
   - Patient/panel populations
   - The public
   - AHS/AH

   Are there gaps, timeliness, credibility/truth issues with any of these communications?
